# Supplementary material for: Comprehensive Annotation and Functional Exploration of MicroRNAs in Lettuce
Source: Front Plant Sci. 2021 Dec 24;12:781836. doi: 10.3389/fpls.2021.781836 (PMC8739914; doi:10.3389/fpls.2021.781836)
Supplement: Supplementary file 2 [file Data_Sheet_8.ZIP › GSM2230747.plot/Lsa-miR319f_Lsat_1_v5_gn_9_99020.1_1730_TPlot.pdf]

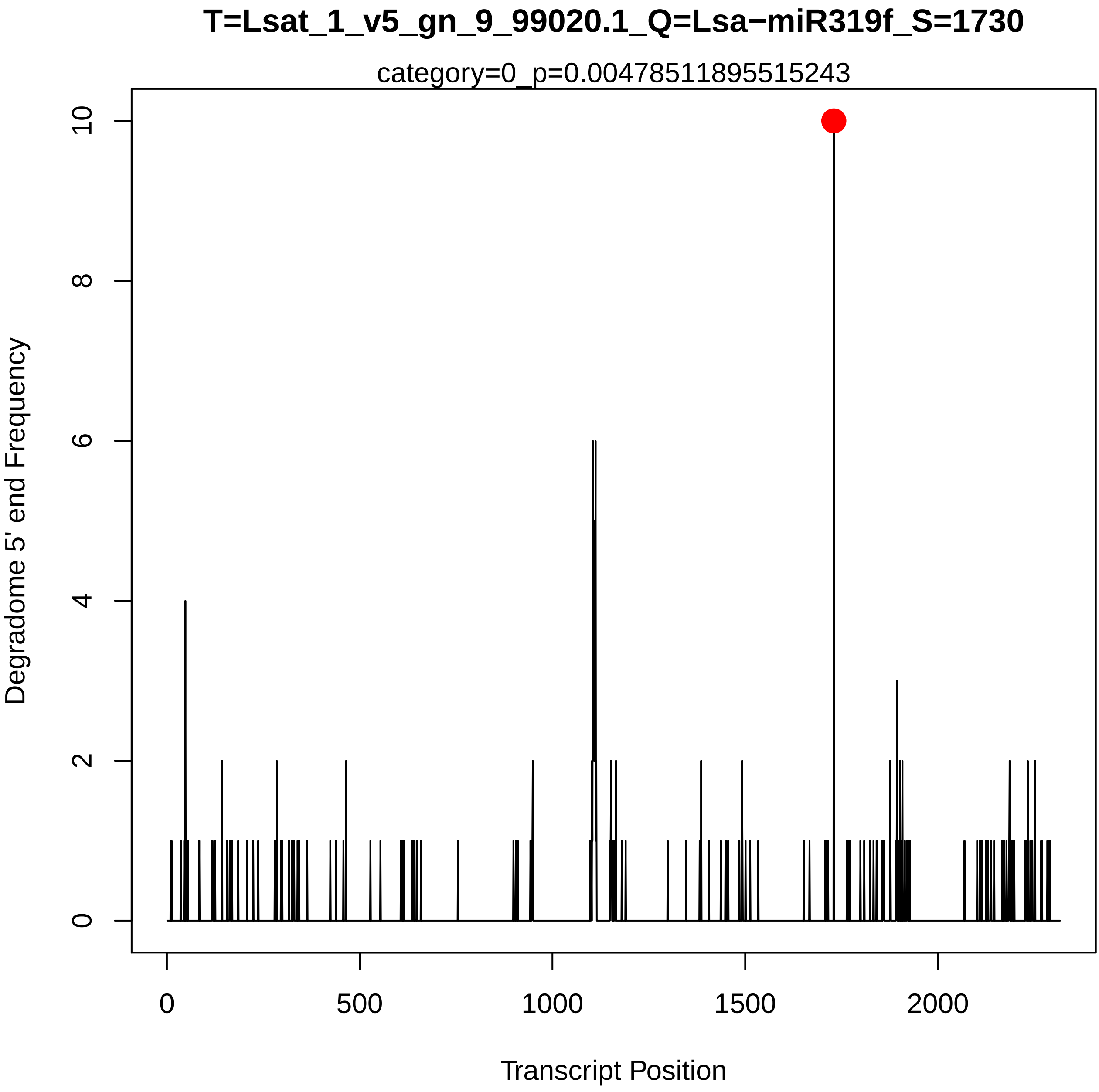

T=Lsat_1_v5_gn_9_99020.1_Q=Lsa−miR319f_S=1730
category=0_p=0.00478511895515243
Degradome 5' end Frequency
0
2
4
6
8
10
0
500
1000
1500
2000
Transcript Position
